# Arterial hemodynamics and its correlation with retinal microarchitecture in pseudoexfoliation glaucoma

**DOI:** 10.5935/0004-2749.20230065

**Published:** 2023

**Authors:** Tolga Kocaturk, Ozge Key Abdullayev, Sinan Bekmez, Yasemin Durum Polat

**Affiliations:** 1 Department of Ophthalmology, Faculty of Medicine, Adnan Menderes University, Aydin, Turkey.; 2 Department of Ophthalmology, Aydin State Hospital, Aydin, Turkey.; 3 Department of Ophthalmology, University of Health Sciences Dr. Behcet Uz Child Disease and Pediatric Surgery Training and Research Hospital, Izmir, Turkey.; 4 Department of Radiology, Faculty of Medicine, Adnan Menderes University, Aydin, Turkey.

**Keywords:** Retinal artery, Tomography, optical coherence, Ophthalmic artery, Nerve fibers, Glaucoma, Ultrasonography, doppler, color, Vascular resistance, Hemodynamics, Retinal ganglion cells, Artéria retiniana, Tomografia de coerência óptica, Artéria oftálmica, Fibras nervosas, Glaucoma, Ultrassonografia doppler em cores, Resistência vascular, Hemodinâmica, Células ganglionares da retina

## Abstract

**Purpose:**

The study aimed to investigate the correlation between arterial hemodynamics measured by color Doppler ultrasonography and retinal microarchitecture parameters determined by spectral-domain optical coherence tomography (SD-OCT) in pseudoexfoliation glaucoma.

**Methods:**

This prospective study included 82 participants. Peripapillary retinal nerve fiber layer, ganglion cell inner plexiform layer, and ganglion cell complex values were measured. Ophthalmic artery and central retinal artery flows were evaluated with color Doppler ultrasonography, and resistivity index values were calculated.

**Results:**

The study included 47 controls and 35 pseudoexfoliation glaucoma cases. In pseudoexfoliation glaucoma group, mean peripapillary retinal nerve fiber layer and ganglion cell complex thickness were statistically significantly lower in all quadrants compared to controls (p<0.001). Resistivity index values of the ophthalmic and central retinal arteries were significantly higher in pseudoexfoliation glaucoma group than in the controls (p<0.001 and r=0.684). Resistivity index values of the ophthalmic and central retinal arteries with ganglion cell complex thickness correlated significantly. On the other hand, no significant relationship for retinal nerve fiber layer thickness was identified.

**Conclusions:**

Structural changes (ganglion cell complex and ganglion cell inner plexiform layer) in patients with pseudoexfoliation glaucoma and early glaucomatous loss showed a significant correlation with changes in ocular vascular hemodynamics. In cases where systemic vascular resistance is increased, ganglion cell complex and ganglion cell inner plexiform layer may not exactly reflect glaucoma state. In such cases, thickness changes in the retinal nerve fiber layer may give more realistic results regarding glaucoma. We have seen that pseudoexfoliation glaucoma-induced structural deterioration and increased resistance in ocular hemodynamics correlated with ganglion cell complex, but not retinal nerve fiber layer.

## Abstract (pt)

**Objetivo:**

Investigar a correlação entre a hemodi­nâmica arterial, medida pela ultrassonografia com Doppler colorido, e os parâmetros de microarquitetura da retina, determinados pela tomografia de coerência óptica de domínio espectral (SD-OCT) no glaucoma pseudoexfoliativo.

**Métodos:**

Foram incluídos 82 participantes neste estudo prospectivo. Foram medidos os valores da camada de fibras nervosas da retina peripapilar, da camada plexiforme interna de células ganglionares e do complexo de células ganglionares. Os fluxos da artéria oftálmica e da artéria central da retina foram avaliados com ultrassonografia por Doppler colorida e foram calculados os valores do índice de resistividade.

**Resultados:**

Foram incluídos no estudo 47 casos de controle e 35 casos de glaucoma pseudoexfoliativo. No grupo com glaucoma pseudoexfoliativo, a média da camada de fibras nervosas da retina peripapilar e a espessura do complexo de células ganglionares foram menores em todos os quadrantes em comparação com os controles, com significância estatística (p<0,001). Os valores do índice de resistividade das artérias oftálmica e central da retina foram significativamente maiores no grupo com glaucoma pseudoexfoliativo que nos controles (p<0,001 e r=0,684). Ao se compararem os valores do índice de resistividade das artérias oftálmica e central da retina com a espessura do complexo de células ganglionares, foi encontrada uma correlação significativa entre elas. Por outro lado, não detectamos uma relação significativa para a espessura da camada de fibras nervosas da retina. **Conclusões:** Alterações estruturais (complexo de células ganglionares, camada plexiforme interna de células ganglionares) em pacientes com glaucoma pseudoexfoliativo com perda glaucomatosa precoce mostraram uma correlação significativa com alterações na hemodinâmica vascular ocular. Nos casos em que a resistência vascular sistêmica é aumentada, o complexo de células ganglionares e a camada plexiforme interna de células ganglionares podem não refletir exatamente o estado do glaucoma. Nesses casos, alterações na espessura da camada de fibras nervosas da retina podem dar resultados mais realistas em relação ao glaucoma. Observou-se uma correlação da deterioração estrutural induzida pelo glaucoma pseudoexfoliativo e do aumento da resistência na hemodinâmica ocular com o complexo de células ganglionares, mas não com a camada de fibras nervosas da retina.

## INTRODUCTION

Pseudoexfoliation (PE) is the first identified cause of open-angle glaucoma worldwide^(1)^. The accumulation of PE material in ocular tissues is a systemic and age-related condition^(2)^. PE in the juxtacanalicular region, increased aqueous protein concentration and cellular dysfunction, and primary functional retinal ganglion cells impairment are possible pathological mechanisms in PE glaucoma (XFG)^(3)^. XFG develops due to blockage of the trabecular meshwork by PE material and is a severe type of glaucoma^(4,5)^.

Optical coherence tomography (OCT) is widely used for diagnosis and follow-up in patients with glaucoma^(6)^. Analyses regarding the peripapillary retinal nerve fiber layer (RNFL) and ganglion cell complex (GCC) can be performed to differentiate glaucomatous damage^(7)^. When OCT technology was first used, evaluation of glaucomatous structural damage was limited to RNFL. However, in later studies, the evaluation of ganglion cell inner plexiform layer (GCIPL) and GCC has been comparable with RNFL parameters, and these measurements are better parameters in glaucoma diagnosis^(8,9)^.

Color Doppler ultrasonography (CDU) is a noninvasive and reproducible method to evaluate ocular hemodynamic parameters^(10)^. PE material has been shown to cause optic disk changes and hemodynamic disorders without high intraocular pressure (IOP)^(11,12)^. It is known that changes in optic nerve head (ONH) blood flow are important for the development and progression of various glaucoma types^(13)^.

This study aimed to examine whether spectral-do­main OCT and CDU findings show any correlation in patients with XFG and compare those patients to healthy subjects.

## METHODS

The study was carried out prospectively and randomized in the Glaucoma Unit of the Department of Ophthalmology, Faculty of Medicine, Adnan Menderes University, Aydin, Turkey, with the ethics committee glaucoma numbered 2018/1400. All participants have read and signed an informed consent form with detailed explanations.

### Subjects

The patients were divided into two groups: XFG and control groups.

Patients diagnosed with XFG with early glaucomatous loss according to Hodapp classification were included in the study. According to this classification, mean deviation (MD) <-6 dB, fewer than 18 points depressed below the 5% probability level and fewer than 10 points below the p<1% level, and no point in the central 5 degrees with a sensitivity of <15 dB were considered an early glaucomatous loss.

Patients with XFG were identified by present glaucomatous optic disk changes with corresponding defects and present PE material on the pupillary rim and/or lens capsule on biomicroscopic examination with and without dilatation and open-angle confirmed by gonioscopic examination (>180° visible pigmented posterior trabecular meshwork on nonindentation gonioscopy in primary position, being grade 3 or higher according to Shaffer classification). The glaucomatous changes in the disk were defined as vertical cup/disk (C/D) ratios greater than 0.6 or the difference between the two eyes greater than 0.2, diffuse or focal rim thinning, notching, excavation, or splinter bleeding near the optic disk consistent with glaucoma. Glaucomatous visual field (VF) defect presence was defined as glaucoma hemifield test outside the normal range, standard pattern deviation with p<5%, or a cluster >3 points in the pattern deviation plot in a single hemifield (superior or inferior) with p<5%, and at least one of these with p<1%. IOP was measured using Goldmann applanation tonometer (GAT). All patients with XFG had at least two IOP readings >21 mm Hg in their previous ophthalmic exams.

Control group inclusion criteria were as follows: PE material absence in the anterior segment examination, IOP below 22 mmHg, glaucomatous optic disk findings absence, open anterior chamber angle (grade 3 and above according to Shaffer classification), glaucomatous VF defect absence, and absence of chronic systemic diseases that could probably affect the eye (hypertension, diabetes mellitus, etc.). If both eyes met the criteria, only one randomly selected eye was included.

Exclusion criteria for both groups were as follows: ambient opacities that interfere with routine ophthalmologic examination (corneal opacity, intense cataract, etc.), having more than 3 diopters of myopia or hypermetropia and more than 1 diopter of astigmatism, any retinal disease, retinal detachment, retinopathy, or maculopathy (senile macular degeneration, diabetic retinopathy, etc.), an advanced systemic disease, a history of ocular trauma, systemic steroid use, contact lens use, or ocular surgery (except for uneventful cataract surgery within 6 months prior to the enrollment), hereditary or acquired pathologies in the optic disk or nonglaucomatous optic neuropathy, chronic systemic diseases that can affect the eye (hypertension, diabetes mellitus, etc.), pregnancy, uveitis history, use of drugs affecting the vascular system, media opacity, including corneal scar, opacity preventing adequate image quality, opacity causing an artifact in OCT measurement, and patients who cannot be measured with OCT device and CDU.

### Ophthalmologic examinations

Detailed ophthalmological examinations were performed by the same ophthalmologist (TK). In the first phase, autorefractometry and IOP measurements (Nidek Tonoref II, Japan), best-corrected visual acuity (BCVA) evaluation (assessed in decimal with Snellen chart and then converted to LogMAR), anterior segment view with slit-lamp biomicroscopy, angle examination with four mirror Sussman Gonioscope Lens (Ocular Instruments, Inc., Bellevue, WA, USA), and dilated fundus examination (with +78 diopter lens) were performed. In the second phase, diurnal IOP measurement with GAT (Haag-Streit International AT 900 Applanation Tonometer mounted on a Topcon SL-1E microscope) and VF test (Humphrey Instruments Model 740; Carl Zeiss, USA using 24-2 SITA standard program) were made.

### OCT imaging

Participants’ images were taken by spectral-domain OCT (Cirrus HD-OCT 5000 Carl Zeiss Meditec, Dublin, CA) using the macular map and disc map protocols with good quality signal strength (≥6). For RNFL measurements, information was collected from a 200 × 200 × 1024 (height × width × depth) point obtained in a 6 × 6 × 2-mm cube centered on the optic nerve and compared with an age-matched normative database. RNFL thickness and ONH parameters were automatically calculated by the device on the same output. GCC was obtained by segmenting an area of 6 × 6 × 2 mm with an ellipse centered on the fovea. Average and minimum GCIPL thicknesses were calculated as sectoral maps in 6 quadrants (superior, superonasal, inferonasal, inferior, inferotemporal, and superotemporal), and other specific parameters, such as deviation maps, were compared with the normative age-matched database. The average and minimum GCIPL thicknesses within the elliptical ring were recorded in the thickness chart seen in the middle of the printout. In addition to ONH parameters, rim area and vertical C/D ratio were also evaluated.

### CDU imaging

All measurements were performed by the same radiologist (YDP) in a double-blind fashion. Ultrasonographic evaluations were performed using Toshiba Applio 80 (Toshiba Medical Systems Corporation, Tochigi, Japan) device. In the first stage, gray-scale examinations were performed on both eyes. In the second stage, RDUS images of the same vessels were obtained. In the third stage, ophthalmic artery (OA) and central retinal artery (CRA) flow samples were obtained. Patients’ measurements were taken while they were lying in the supine position after resting for 15 minutes in the examination room at normal room temperature. Patients were told to keep their eyes closed during the examination and not to move their eyes unless otherwise indicated. The probe, on which methylcellulose gel was applied, was placed on the eyelids, and the measurement was made. Care was taken to not apply excessive pressure to the eyeball to avoid artifacts and an effect on vascular resistance. Medium- and high-color mode gain settings were used, and artifacts due to involuntary eye movements were reduced. Gray-scale images were produced first to serve as an anatomical reference. Then, CDU was employed to characterize blood flows in OA and CRA. Specifically, peak systolic velocity (PSV, cm/s) and end-diastolic velocity (EDV, cm/s) were measured at the intersection of OA with the optic nerve. In OA axial and oblique plans, parallel to the long axis and Doppler angles were evaluated to be between 45° and 60°. CRA measurements were taken from the anterior optic nerve shadow part. Then, the resistivity index (RI) value formula RI = (PSV - EDV)/PSV defined by Pourcelet was calculated.

### Statistical analysis

SPSS Windows 22.0 program was used for statistical analysis. Whether the parameters had a normal distribution was checked by Kolmogorov-Smirnov test. Student t-test, one-way analysis of variance, and Pearson correlation analysis were used for normally distributed data analysis, while Mann-Whitney U test, Kruskal-Wallis analysis, and Spearman correlation analysis were used for not normally distributed data analysis. Qualitative variables descriptive statistics was specified as n (%). Statistical significance was evaluated as p<0.05. Receiver operation characteristic (ROC) was analyzed to determine the value of RI measurements derived from CDU for XFG diagnosis.

## RESULTS

The study included 35 eyes of 35 patients with XFG and 47 eyes of 47 healthy patients (82 patients in total). Bilaterality was observed in 21 patients (60%) in XFG group. Patients’ eyes to be included in the study were determined randomly. There was no statistically significant difference between patients with XFG and healthy subjects in terms of age (68.3 ± 6.79 and 67.4 ± 6.29 years, respectively, p=0.530). Gender ratio (male/female) was 22/13 and 27/20 in patients with XFG and healthy subjects, respectively (p=0.655). IOP values in the two groups were 16.6 ± 3.98 and 15.3 ± 2.82 mmHg, respectively (p=0.121). BCVA was higher in control group than in XFG group (0.04 ± 0.88 and 0.06 ± 0.95 LogMAR, respectively, p=0.097).

### OCT findings

GCC and RNFL thickness values were statistically lower in all quadrants in XFG group than in control group (p<0.001) (Table 1). In both groups, a statistically significant negative correlation was found between age and GCC (Table 2). When the relationship of RNFL thickness with age was examined, no statistically significant relationship was found in control group. However, in XFG group, there was a weak negative correlation with age only in nasal quadrant thickness (r=-0.344 and p=0.009). No statistically significant correlations were observed regarding the relationship between age, rim area, and vertical C/D ratios in both groups.

**Table 1:** Comparison of RNFL, ONH, and GCC parameters between the two groups

	XFG Group (mean ± SD, µm)	Control Group (mean ± SD, µm)	p-value*
Average RNFL	74.7 ± 15.9	92.1 ± 7.9	**<0.001**
Superior RNFL	90.3 ± 20.8	114.7 ± 13.6	**<0.001**
Inferior RNFL	92.9 ± 27.9	117.0 ± 13.5	**<0.001**
Nasal RNFL	63.8 ± 11.9	72.3 ± 11.1	**<0.001**
Temporal RNFL	52.2 ± 12.4	65.6 ± 10.4	**<0.001**
Rim Area (mm^2^)	1.07 ± 0.36	1.39 ± 0.27	**<0.001**
Vertical C-D ratio	0.60 ± 0.19	0.48 ± 0.17	**<0.001**
Average GCIPL	68.5 ± 12.3	81.4 ± 5.67	**<0.001**
Minimum GCIPL	62.7 ± 14.7	78.0 ± 6.4	**<0.001**
Superior-Nasal GCC	69.5 ± 13.0	82.7 ± 6.5	**<0.001**
Superior GCC	68.6 ± 13.1	82.4 ± 6.11	**<0.001**
Superior-Temporal GCC	68.8 ± 11.9	80.3 ± 5.5	**<0.001**
Inferior-Nasal GCC	69.3 ± 12.7	81.0 ± 6.5	**<0.001**
Inferior GCC	68.1 ± 13.2	79.8 ± 6.2	**<0.001**
Inferior-Temporal GCC	68.6 ± 12.3	81.8 ± 5.8	**<0.001**

SD= standard deviation; OCT= optical coherence tomography; RNFL=retinal nerve fiber layer; XFG= pseudoexfoliation glaucoma; GCIPL= ganglion cell inner plexiform layer; GCC= ganglion cell complex; ONH= optic nerve head.

* Independent sample t-test, statistically significant difference p<0.05.

**Table 2:** Correlation of RNFL and GCC thicknesses in the two groups with age

	XFG group	Control group
Average GCIPL-Age	**r=-0.372** p=0.005	r=-0.181 p=0.081
Minimum GCIPL-Age	**r=-0.343** **p=0.010**	**r=-0.283** **p=0.006**
Superior-Nasal GCC-Age	**r=-0.374** **p=0.004**	**r=-0.319** **p=0.002**
Superior GCC-Age	**r=-0.353** **p=0.008**	**r=-0.247** **p=0.017**
Superior-Temporal GCC-Age	**r=-0.346** **p=0.009**	**r=-0.278** **p=0.007**
Inferior-Nasal GCC-Age	**r=-0.291** **p=0.030**	**r=-0.270** **p=0.009**
Inferior GCC-Age	**r=-0.365** p=0.006	r=-0.189 p=0.068
Inferior-Temporal GCC-Age	**r=-0.367** **p=0.005**	r=-0.194 p=0.060
Average RNFL-Age	r=-0.257 p=0.056	r=-0.039 p=0.708
Superior RNFL-Age	r=-0.240 p=0.075	r=-0.070 p=0.502
Inferior RNFL-Age	r=-0.172 p=0.205	r=-0.082 p=0.430
Nasal RNFL-Age	**r=-0.344** **p=0.009**	r=-0.013 p=0.903
Temporal RNFL-Age	r=-0.089 p=0.51	r=0.095 p=0.364

OCT= optical coherence tomography; GCIPL= ganglion cell inner plexiform layer; GCC= ganglion cell complex; XFG= pseudoexfoliation glaucoma.

* Spearman correlation analysis, statistically significant difference p<0.05

### CDU findings

Mean OA-RI values in patients with XFG and healthy controls were 0.75 ± 0.06 and 0.68 ± 0.04, respectively (p<0.001). Mean CRA-RI values in patients with XFG and healthy controls were 0.68 ± 0.05 and 0.64 ± 0.04, respectively (p<0.001). A positive and moderate-sized correlation was observed in Pearson correlation test for OA and CRA-RI values in XFG group (r=0.684), and a statistically significant correlation was observed in XFG group, unlike in control group (p<0.001). In control group, a statistically significant, moderate-sized, and positive correlation was detected in the relationship between OA and CRA-RI (r=0.625 and p<0.001) using Spearman correlation test.

### Correlation between arterial hemodynamics and retinal structure

There was no significant correlation between OA-RI and peripapillary RNFL thickness values in XFG group (Table 3). However, a small, negative, and statistically significant correlation was observed with mean, minimum, inferior, superior, and superonasal GCC thicknesses.

**Table 3:** Correlation of RNFL and GCC thicknesses in the two groups with OA RI

	XFG group	Control group
Average GCIPL-RI-OA	**r=-0.269** p=0.045	r=-0.070 p=0.500
Minimum GCIPL-RI-OA	**r=-0.283** p=0.034	r=-0.132 p=0.205
Superior-Nasal GCC-RI-OA	**r=-0.322** p=0.016	r=-0.192 p=0.064
Superior GCC-RI-OA	**r=-0.300** p=0.025	r=-0.087 p=0.404
Superior-Temporal GCC-RI-OA	r=-0.197 p=0.145	r=-0.051 p=0.612
Inferior-Nasal GCC-RI-OA	r=-0.239 p=0.077	r=-0.195 p=0.074
Inferior GCC-RI-OA	**r=-0.273** p=0.047	r=-0.144 p=0.165
Inferior-Temporal GCC-RI-OA	r=-0.231 p=0.087	r=-0.102 p=0.327
Average RNFL-RI-OA	r=-0.108 p=0.299	r=-0.048 p=0.723
Superior RNFL-RI-OA	r=-0.144 p=0.165	r=-0.037 p=0.787
Inferior RNFL-RI-OA	r=0.100 p=0.464	r=-0.058 p=0.577
Nasal RNFL-RI-OA	r=-0.206 p=0.128	r=-0.051 p=0.623
Temporal RNFL-RI-OA	r=0.066 p=0.631	r=0.130 p=0.213

OCT= optical coherence tomography; GCIPL= ganglion cell inner plexiform layer; GCC= ganglion cell complex; RNFL= retinal nerve fiber layer; OA-RI= ophthalmic artery-resistivity index; XFG= pseudoexfoliation glaucoma.

* Pearson correlation analysis, statistically significant difference p<0.05.

No statistically significant correlation was found between CRA-RI and peripapillary RNFL thickness in XFG group. However, a significant, negative, and small effect-size correlation in mean, minimum, superonasal, inferotemporal, and inferior GCC thickness values was observed (Table 4).

**Table 4:** Correlation of RNFL and GCC thicknesses in the two groups with CRA-RI

	XFG Group	Control Group
Average GCIPL-RI-CRA	**r=-0.298** p=0.025	r=-0.049 p=0.638
Minimum GCIPL-RI-CRA	**r=-0.335** p=0.012	r=-0.094 p=0.369
Superior-Nasal GCC-RI-CRA	**r=-0.295** p=0.027	r=-0.064 p=0.540
Superior GCC-RI-CRA	r=-0.247 p=0.066	r=-0.125 p=0.230
Superior-Temporal GCC-RI-CRA	r=-0.197 p=0.145	r=-0.093 p=0.371
Inferior-Nasal GCC-RI-CRA	r=-0.253 p=0.060	r=-0.135 p=0.195
Inferior GCC-RI-CRA	**r=-0.293** p=0.028	r=-0.092 p=0.378
Inferior-Temporal GCC-RI-CRA	**r=-0.287** p=0.032	r=-0.024 p=0.818
Average RNFL-RI-CRA	r=-0.075 p=0.584	r=-0.070 p=0.500
Superior RNFL-RI-CRA	r=-0.198 p=0.056	r=-0.115 p=0.399
Inferior RNFL-RI-CRA	r=-0.046 p=0.736	r=0.038 p=0.714
Nasal RNFL-RI-CRA	r=-0.129 p=0.342	r=-0.186 p=0.073
Temporal RNFL-RI-CRA	r=0.030 p=0.826	r=0.197 p=0.058

OCT= optical coherence tomography; GCIPL= ganglion cell inner plexiform layer; GCC= ganglion cell complex; RNFL= retinal nerve fiber layer; CRA-RI= central retinal artery-resistivity index; XFG= pseudoexfoliation glaucoma.

* Pearson correlation analysis, statistically significant difference p<0.05.

### ROC analysis

**Figure 1:**
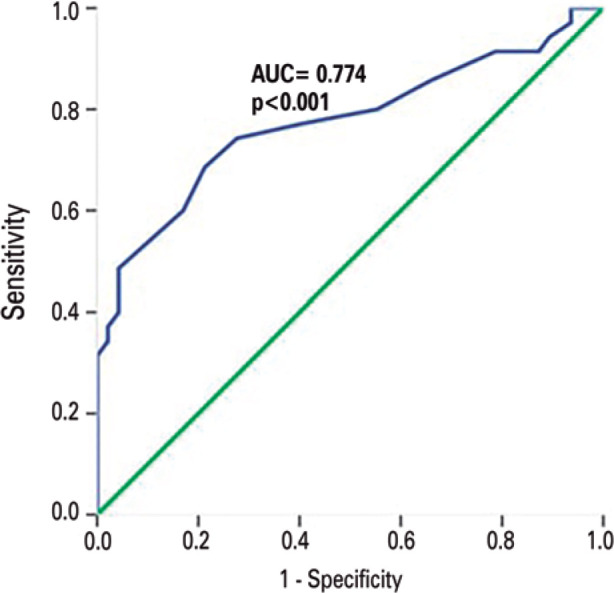
Receiver operation characteristic of resistivity index for ophthalmic artery.

**Figure 2:**
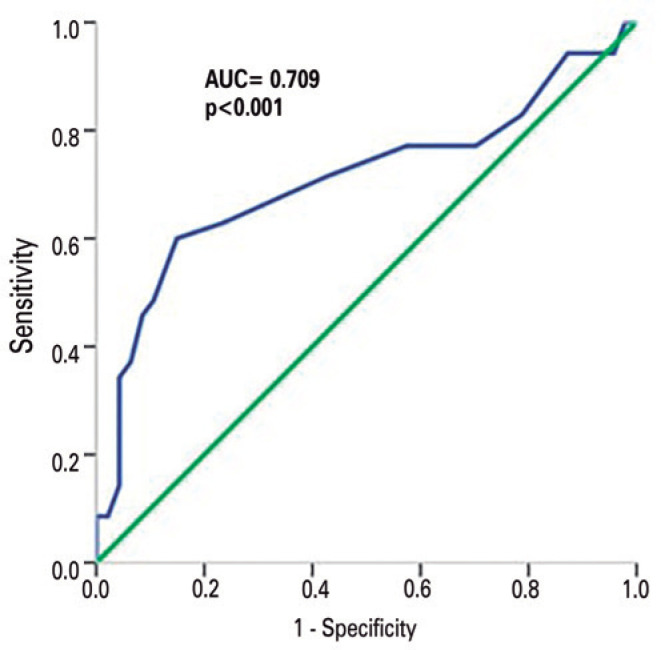
Receiver operation characteristic of resistivity index for central retinal artery.

RI-OA and RI-CRA use can potentially be valuable biomarkers in the early diagnosis of XFG provided that threshold values under certain criteria can be determined for diagnostic decision making. For this purpose, ROC analysis was carried out on these parameters, and the graphs in figures 1 and 2 were produced.

## DISCUSSION

Glaucoma is a progressive optic neuropathy that occurs in all retinal ganglion cells segments^(14)^. Especially compared with primary open-angle glaucoma, patients with XFG are characterized by high mean IOP, wide fluctuation range, severe VF damage, retrobulbar hypoperfusion, and rapid disease progress^(15)^. The inclusion of many factors, such as IOP value, rim area, C/D ratio, VF parameters, RNFL, and GCC thickness values while evaluating patients with suspected glaucoma or diagnosed with glaucoma provides very important information about disease progression^(16)^. In our study, the correlation between GCC and peripapillary RNFL thickness and ONH parameters with CDU measurements in patients with XFG were evaluated and compared with normal cases.

Patients with early-stage glaucoma have optic disc deterioration before VF deterioration occurs. Studies have shown abnormal RNFL findings 5 years before VF defect appearance in more than 60% of patients; therefore, ganglion cells should be examined early during the progression of exfoliation syndrome to XFG^(17)^. Studies have suggested that XFG progression can make RNFL thinner^(17,18)^. In XFG group, GCC and peripapillary RNFL thicknesses were lower in all quadrants. In ONH examinations, vertical C/D ratio was higher in XFG group compared to control group, and the rim area was lower. Our OCT data were compatible with the literature^(18)^. The results showed that OCT has a high ability to distinguish between normal participants and patients with glaucoma. In glaucoma evaluation based on OCT, it is generally accepted as the best approach to evaluate RNFL and GCC together^(19)^. However, in cases where there are intense media opacities, or in some retinal diseases, such as advanced myopia, OCT cannot obtain sufficient quality measurements. Also, in such cases, IOP measurement and VF test may not provide reliable information.

CDU is a noninvasive tool used to measure blood flow values in various anatomical regions. The blood flow in ONH has been shown to be impaired in glaucoma^(20)^. It has been reported that CDU, which is used to evaluate ocular blood flow, can be used safely in patients with glaucoma diagnosis and follow-up^(21)^.

Increased vascular bed resistance causes EDV to decrease more than PSV. This situation calculated using Pourcelot formula, results in a high RI value. The RI shows the resistance to blood flow in the peripheral vascular bed and can be used to assess organ perfusion^(22)^. With this study, we showed that OA-RI and CRA-RI values were significantly higher in XFG group than in healthy controls. It has been previously shown that retrobulbar hemodynamics worsens in this patient group^(11,12,23)^. Tiwari et al.^(24)^ found high RI values in both primary open-angle and normotensive glaucoma. Risk factors independent of IOP, such as impaired ocular/retrobulbar perfusion, which are more prominent in XFG, may further increase glaucomatous damage rate seen in XFG^(25)^. We did not come across a detailed study in the literature that aimed to investigate whether there is a correlation between how much hemodynamic disturbances, which are evident in XFG, affect glaucoma progression or how much the parameters are used to determine glaucoma progression. We found no significant correlation between OA-RI/CRA-RI and peripapillary RNFL thickness and ONH parameters in XFG group. However, in XFG group, we found a significant relationship between OA-RI and mean, minimum, superonasal, superior, and inferior GCC thickness. Similarly, we showed that there is a negative correlation between CRA-RI and mean, minimum, superonasal, inferotemporal, and inferior GCC thickness.

In the pathogenesis related to blood flow in glaucoma, ischemic periods caused by vasospastic diseases and paroxysmal changes are at the forefront. It is known that glaucoma is associated with systemic vascular blood flow disorders and vasospasm, similar to migraine^(26)^. Permanent blood flow changes in various brain parts have been reported to accompany migraine, and similarly, changes in optic nerve hemodynamics have been observed in glaucoma^(27)^. The risk of developing glaucoma may increase in patients with migraine^(28)^. In a study, it was found that migraine and high IOP increa­se the risk of low mean ocular perfusion pressure, and this may have a causal relationship with impaired ONH blood flow^(29)^. In another study, lamina cribrosa and RNFL thicknesses were lower in patients with migraine, and disease duration was significantly correlated with RNFL thickness^(30)^.

It is still unclear whether the changes in retinal nerve cells cause vascular changes or vascular changes cause nerve cell layer thinning.

However, increased vascular resistance in OA and CRA parts in patients with XFG and the fact that these increases are in parallel with OCT findings suggest that PE-caused glaucoma has systemic causes besides IOP increase. If this situation is demonstrated with further studies, it may be necessary to add systemic agents that reduce vascular resistance to XFG therapy in the future. From another point of view, the correlation of CDU findings with OCT findings suggests that OCT findings may also be correlated with increased systemic vascular resistance. Since GCC may be affected in cases that increase vascular resistance due to a systemic disease or systemic drug use, GCC values, in such cases, may not fully reflect glaucoma state. Considering that RNFL is not affected by changes in RI, it is expected that RNFL thickness measurements will give more realistic results regarding glaucoma when systemic vascular resistance is increased.

The limitation of our study is that it only included patients with XFG. Our results may not be valid for other open-angle glaucoma types. It should be investigated whether CDU and OCT findings correlate in the same way in different glaucoma types. In this direction, we also have planned new studies, and they are progressing. Important strengths of our study are its prospective nature and our recording of only early-stage XFG cases.

In conclusion, this study demonstrated a significant correlation between GCC parameters and increased arterial blood flow resistance in patients with XFG and early glaucomatous period. However, no relationship was found between peripapillary RNFL changes and arterial blood flow alterations. These findings suggest that the pathophysiology of the changes seen in peripapillary RNLF and macular GCC may be different. Further studies are needed to reveal the details about the associations between hemodynamics and its reflections on retinal microarchitecture.
